# Hypertriglyceridemic Pancreatitis Treated with Insulin Therapy: A Comparative Review of 34 Cases

**DOI:** 10.7759/cureus.3501

**Published:** 2018-10-27

**Authors:** Faisal Inayat, Fahad Zafar, Asad S Baig, Najaf A Chaudhry, Aysha Aslam, Zarak H Khan, Muhammad J Iqbal

**Affiliations:** 1 Internal Medicine, Allama Iqbal Medical College, Lahore, PAK; 2 Internal Medicine, King Edward Medical University, Lahore, PAK; 3 Internal Medicine, Nawaz Sharif Medical College, Gujrat, PAK; 4 Internal Medicine, Lahore Medical and Dental College, Lahore, PAK; 5 Internal Medicine, Beth Israel Deaconess Medical Center, Boston, USA; 6 Internal Medicine, St. Mary Mercy Hospital, Livonia, USA

**Keywords:** hypertriglyceridemia, acute pancreatitis, insulin therapy, diagnosis, treatment, clinical outcomes

## Abstract

Although the clinical presentation of hypertriglyceridemic pancreatitis is usually similar to other forms of acute pancreatitis, it is frequently associated with increased clinical severity and rate of complications. Therefore, appropriate and timely management is of paramount importance in these patients. We performed a structured literature search of the medical databases PubMed and Google Scholar, using the terms “hypertriglyceridemia,” “acute pancreatitis,” “insulin,” and “treatment.” In this search, we identified 34 cases of hypertriglyceridemia-related pancreatitis available in the full-text form in English. The data on patients’ characteristics, epidemiology, clinical features, comorbid conditions, and diagnostic modalities were collected and summarized. This review illustrates that the use of insulin therapy with close monitoring of blood glucose levels is safe. It can be considered as an important component of management in patients with hypertriglyceridemia-related pancreatitis, especially in a clinical setting without the availability of plasmapheresis. Randomized clinical trials are warranted to outline a generalized and efficient treatment regimen for hypertriglyceridemic pancreatitis.

## Introduction and background

Acute pancreatitis is a common inflammatory condition of the pancreas with various underlying etiologies. Gallstones and alcohol are among the most common while drug-induced disease, autoimmune conditions, viral infections, trauma, and malignancy are less common causes [1-3]. However, relatively infrequent hypertriglyceridemia is one of the known etiologies of acute pancreatitis [4]. In patients with hypertriglyceridemic pancreatitis (HTGP), a pre-existing lipid abnormality has commonly been documented. According to prior research, the risk of acute pancreatitis increases significantly when serum hypertriglyceridemia exceeds 1,000 mg/dL (normal, 101-150 mg/dL) [5]. The concomitant presence of single or multiple secondary precipitating factors, including poorly controlled diabetes mellitus, alcohol consumption, or therapy with certain medications, are also implicated in the pathogenesis of HTGP. Similarly, genetic factors may also be deemed responsible for altering the normal lipid metabolism in these patients [6].

The overall mortality following acute pancreatitis has been described up to 5%; however, it is relatively higher among hospitalized patients [6]. HTGP frequently culminates in a severe disease presentation with an increased propensity to cause life-threatening complications. In this context, an effective and feasible treatment of choice is particularly warranted. The major goal of therapy is to relieve pain and avoid any inadvertent events. In the current times, a specific therapeutic approach is non-existent. Conservative management, using the variable combinations of intravenous hydration, analgesics, and antibiotics, is important for the initial resuscitation. Although heparin and insulin have been used, plasmapheresis and lipid apheresis are becoming more popular management options for hyperlipidemic pancreatitis [7]. However, the widespread availability of these techniques may pose a therapeutic dilemma. This review outlines our current understanding of the epidemiology and risk factors for HTGP, the pathophysiology of this disorder, and currently available approaches to diagnosis and treatment. We highlight insulin therapy as a feasible therapeutic option in these patients, especially if plasmapheresis is not available.

## Review

Epidemiology

HTGP is a known clinical entity with an estimated incidence ranging from 2.3% to 10% of all cases of acute pancreatitis [7]. It often involves male individuals with age less than 50 years who have severe hypertriglyceridemia. According to a prospective study of 400 cases of acute pancreatitis, patients with hypertriglyceridemia were younger, obese, predominantly of the male gender, diabetic, and had a history of more frequent episodes of persistent organ failure [8]. Furthermore, hypertriglyceridemia is commonly encountered in pregnancy due to physiological changes and, occasionally, it may lead to acute pancreatitis. The incidence of HTGP during pregnancy has been described to be 1 in 25,000 pregnancies.

Pathogenesis

Although the exact pathogenesis of HTGP is unknown, the proposed mechanism implicates the toxic effects of free fatty acids released by the breakdown of triglycerides [9]. Triglyceride-rich lipoproteins such as chylomicrons and very low-density lipoproteins (VLDLs) are large molecules that are found in abundance in conditions like hypertriglyceridemia. These molecules may occlude the pancreatic capillaries and subsequently change the structure of the acinar cells. This process eventually triggers the release of pancreatic enzymes, including lipases that catabolize the lipid-rich molecules. This breakdown results in increased local oxidative stress that further contributes to the inflammatory response in the pancreas, resulting in the symptomatology of acute pancreatitis [9].

Search criteria

We conducted an extensive literature search of Google Scholar and PubMed (National Library of Medicine, Bethesda, MD) using a combination of terms, including “hypertriglyceridemia,” “pancreatitis,” “insulin,” and “treatment.” A total of 147 studies were initially obtained, consisting of but not limited to original articles, case series, and case reports as of July 2018. After excluding 39 duplicate articles, 108 papers were thoroughly studied. The articles available in any language other than the English were excluded from the review. Forty-eight papers were found relevant to the scope of our study but 34 cases were found accessible in order to retrieve pertinent data required for this review [10-31]. The data on individual cases of HTGP on epidemiology, clinical presentation, diagnosis, management, and clinical outcomes are summarized in Table 1.

**Table 1:** Literature review of insulin treatment in patients with hypertriglyceridemia-associated acute pancreatitis TG, triglyceride; HTG, hypertriglyceridemia; OCPs, oral contraceptive pills; DM, diabetes mellitus; U, unit; D5W, dextrose 5% in water; HTN, hypertension; HLD, hyperlipidemia; CD, Crohn’s disease; MI, myocardial infarction; CABG, coronary artery bypass graft; RE, reflux esophagitis; BD, bipolar disorder; IBS; irritable bowel syndrome; GAD, generalized anxiety disorder; AP; acute pancreatitis; DKA; diabetic ketoacidosis; OSA, obstructive sleep apnea; NA, not available *After acute phase of insulin treatment

Authors	Age/Gender	Clinical presentation	Comorbidity	Initial TG level (mg/dL)	Treatment	TG post-treatment (mg/dL)	Duration of treatment (days)	Outcome
Jabbar et al. 1998 [10]	13/M	Abdominal pain	HTG	4574	Regular insulin injection (0.1 U/kg/h), fish oil, gemfibrozil	565*	1	Recovered
Huang et al. 2002 [11]	41/F	Abdominal pain, nausea, vomiting	HTG, OCPs	2260	Insulin-glucose infusion	314	3	Recovered
Mikhail et al. 2005 [12]	38/F	Excruciating abdominal pain	HTG	10, 560	Subcutaneous insulin lispro, sliding scale	712	3	Recovered
Alagozlu et al. 2006 [13]	44/M	Abdominal pain, vomiting	DM type 2	1707	Regular insulin 3 U/h, D5W, 5000 U of heparin	180	10	Recovered
Gursoy et al. 2006 [14]	24/F	Severe abdominal pain, fever, vomiting, malaise	Pregnancy	10,092	Intravenous insulin, glucose	608	5	Recovered
Jain et al. 2007 [15]	55/M	Abdominal pain, vomiting	Recurrent acute pancreatitis	1808	Regular insulin infusion, D5W, 5000 U heparin subcutaneously twice a day	325	5	Recovered
Jain et al. 2007 [15]	46/M	Abdominal pain, vomiting	DM, recurrent pancreatitis	3743	Regular insulin infusion, D5W, 5000 U heparin subcutaneously twice a day	350	5	Recovered
Love et al. 2009 [16]	34/F	Abdominal pain, nausea, vomiting, diarrhea	HTN, HLD, CD, cholecystectomy	10,039	Sliding scale insulin, gemifibrozil	646	9	Recovered
Jain et al. 2009 [17]	54/M	Acute epigastric pain	DM, HTG	10,320	Regular insulin infusion, D5W, heparin	1386	6	Recovered
Hahn et al. 2010 [18]	20/F	Epigastric pain, vomiting, diarrhea	DM type 1	15,240	Continuous insulin infusion, 3% NaCl solution for pseudohyponatremia	309	22	Recovered
Twilla et al. 2012 [19]	39/M	Abdominal pain, nausea, and vomiting	HTG	5366	Continuous insulin infusion and subcutaneous heparin	717	5	Recovered
Patel et al. 2012 [20]	42/F	Abdominal pain, vomiting	DM type 2, type 5 HTG	>5000	Insulin infusion 0.1 U/kg/h, D5W, heparin 600 U/h	923	1	Recovered
Weston et al. 2013 [21]	46/M	Abdominal pain, eruptive xanthomas arms and torso	2 MIs status post CABG surgery and stenting	3026	Insulin-heparin infusion	NA	NA	Recovered
Denecker et al. 2013 [22]	23/F	Epigastric pain, nausea	DM type 2, RE	12,851	Intravenous fluid, insulin, analgesics	549	12	Recovered
Coskun et al. 2015 [23]	41/M	Abdominal pain, nausea, vomiting	HTG	1118	Regular insulin infusion, D5W	355	3	Recovered
Coskun et al. 2015 [23]	48/F	Abdominal pain, nausea, vomiting	DM	1176	Regular insulin infusion, D5W	464	3	Recovered
Coskun et al. 2015 [23]	54/M	Abdominal pain, nausea, vomiting	HTG	1228	Intravenous regular insulin infusion in D5W	489	3	Recovered
Coskun et al. 2015 [23]	35/M	Abdominal pain, vomiting	DM	1027	Regular insulin infusion, D5W	496	3	Recovered
Coskun et al. 2015 [23]	43/F	Abdominal pain, nausea, vomiting	HTG	1004	Intravenous regular insulin infusion in D5W	476	3	Recovered
Coskun et al. 2015 [23]	30/F	Abdominal pain, nausea, vomiting	HTG	1086	Intravenous regular insulin infusion in D5W	481	3	Recovered
Coskun et al. 2015 [23]	59/M	Abdominal pain, nausea, vomiting	DM	1130	Regular insulin infusion, D5W	373	5	Recovered
Coskun et al. 2015 [23]	46/M	Abdominal pain, nausea, vomiting	DM	1156	Intravenous regular insulin infusion in D5W	498	3	Recovered
Coskun et al. 2015 [23]	40/F	Abdominal pain, vomiting	DM	1124	Regular insulin infusion, D5W	494	3	Recovered
Coskun et al. 2015 [23]	45/M	Abdominal pain, nausea, vomiting	DM	1235	Intravenous regular insulin infusion in D5W	276	5	Recovered
Coskun et al. 2015 [23]	65/M	Abdominal pain, vomiting	DM	1190	Intravenous regular insulin infusion in D5W	356	5	Recovered
Coskun et al. 2015 [23]	46/M	Abdominal pain, nausea, vomiting	DM	1215	Regular insulin infusion, D5W	298	5	Recovered
Franco et al. 2015 [24]	50/M	Severe epigastric pain	BD, HLD, DM type 2, obesity, IBS, GAD	3590	Insulin infusion	684	3	Recovered
Khan et al. 2015 [25]	44/F	Abdominal pain, vomiting	HTN	3525	Insulin infusion	675	3	Recovered
Singla et al. 2015 [26]	19/M	Epigastric pain, nausea, polyuria	Obesity, DM	4009	Insulin infusion 6 U/h, D5W, fenofibrate	180	1	Recovered
Amin et al. 2015 [27]	40/F	Abdominal pain, vomiting, diarrhea	Graves diseases, HTG, pregnancy	4106	Low-dose insulin on sliding scale	885	8	Recovered
Abraham et al. 2015 [28]	24/F	Abdominal pain, nausea, vomiting	Asthma, OCP Estrostep	2200	Insulin infusion, omega-3 fatty acids, gemifibrozil	355	7	Recovered
Aryal et al. 2016 [29]	31/M	Epigastric pain	Obesity, HLD, HTN, DM type 2	15,215	Regular insulin (0.4 U/kg/h), heparin infusion	363	6	Recovered
Jeon et al. 2017 [30]	28/F	Epigastric pain	HLD, Pregnancy	10,392	Insulin injection	No improvement	NA	Died (cardiac arrest after AP-related DKA)
Chaudhary et al. 2017 [31]	44/M	Epigastric pain, vomiting	DM, HTG, HTN, obesity, OSA	6,672	Insulin infusion 0.1 u/kg/h, D5W 75 cc/h	500	8	Recovered

Patient demographics and clinical presentations

In the present review, HTGP involved all age groups, with the mean age of 39.6 years (range: 13-65 years). There was no clear gender preponderance (male, n = 18; female, n = 16). The typical presentation was acute-onset abdominal pain while other notable symptoms included nausea and vomiting. Cutaneous signs of hypertriglyceridemia such as eruptive xanthomas over the extensor surfaces of the arms, legs, and buttocks were also noted in a few patients. A majority of patients had comorbid conditions and risk factors like moderate-to-severe hypertriglyceridemia, hypertension, diabetes mellitus, dyslipidemias, pregnancy, alcohol abuse, oral contraceptive use, and obesity. It was an interesting observation that the initial triglyceride levels causing acute pancreatitis were more than 1000 mg/dL in all cases included in this review. It was further notable that there were a few cases where HTGP occurred in pregnant patients. Although pregnancy is associated with an overall increase in the serum triglyceride level, it rarely exceeds 300 mg/dL in most pregnant females.

Diagnosis

The diagnostic criteria for HTGP include the presence of at least two out of three following findings: (a) acute-onset severe epigastric pain radiating to the back; (b) serum lipase or amylase elevated three or more times the upper reference limit; and (c) the three characteristic findings of acute pancreatitis on imaging investigations such as computed tomography, magnetic resonance imaging, or transabdominal ultrasonography [32]. Another major clue for the diagnosis of HTGP includes biochemical evaluation remarkable for serum triglyceride levels more than 1000 mg/dL. The risk factors for hypertriglyceridemia are obesity, pregnancy, family history of hypertriglyceridemia, poorly-controlled diabetes mellitus, and alcoholism[32]. It is important to mention here that a thorough clinical history, including family history of lipid metabolic aberrations and physical examination to identify eruptive xanthomas, can help to channelize the biochemical and radiological investigations toward a timely etiology establishment.

Insulin therapy

Currently, there are no clear therapeutic guidelines for HTGP. Insulin therapy has previously been used in these patients as a minimally invasive and economical strategy with promising outcomes [33-34]. The mechanism by which insulin lowers the level of serum triglycerides is by triggering the enzymatic activity of lipoprotein lipase and inhibition of hormone-sensitive lipase. Lipoprotein lipase metabolizes chylomicrons and VLDLs into the free fatty acids and glycerol. Therefore, it ultimately decreases the serum triglyceride levels. Decreasing the activity of hormone-sensitive lipase causes decreased adipocyte-triglyceride breakdown, resulting in a decreased release of free fatty acids into the circulation, which controls the toxic effects on the pancreas, limiting its active inflammation [35]. In this review, the initial symptomatic management comprised of bowel rest, intravenous fluids, and analgesics. In regard to the emergency management of HTGP, insulin infusion as monotherapy or part of a combination regimen was the most effective option in settings where plasmapheresis was not available or as an alternative approach for patients who could not tolerate apheresis. It was usually given intravenously at a rate of 0.1-0.3 units/kg/hour. Serum triglyceride levels were monitored every 12 hours. With insulin therapy, it was pivotal to measure blood glucose levels and adjuvant 5% dextrose infusion was required when the blood glucose level fell below 200 mg/dL.

Clinical outcomes

According to the results of the outcome analysis of this review, the overall prognosis of HTGP was good with a vast majority of patients recovering completely with intensive insulin therapy. It is quite reassuring that in emergency clinical settings, intravenous insulin was used as a salvage therapy, even in patients with severe HTGP, resulting in a remarkable recovery. In three to five days of insulin therapy, most patients demonstrated a good clinical response; serum triglyceride levels decreased to less than 500 mg/dL after which the treatment was discontinued. A number of patients received insulin as a combination therapy with lipid-lowering drugs. In our review, one patient died from HTGP due to cardiac arrest, which was thought to occur as a sequel to severe metabolic acidosis and electrolyte imbalance. This fatal outcome re-emphasizes that patients with HTGP are more likely to encounter organ failure as compared to the other causes of pancreatitis. Therefore, urgent and appropriate management is essentially important.

Long-term management

Patients with HTGP clinically improve when the serum triglyceride levels fall below 500 mg/dL. However, in order to prevent recurrent episodes and subsequent complications of HTGP, long-term management is warranted to maintain the level of triglycerides below 200 mg/dL. It is particularly tailored to lifestyle modifications with dietary fat and sugar restriction, aerobic exercises, weight loss, and blood sugar control. The hypolipidemic medications like gemfibrozil and fenofibrate lower serum triglyceride levels and reduce the recurrence risk of HTGP [36-37].

Future directions

A prospective randomized controlled trial showed that early high-volume hemofiltration can lower triglyceride levels more efficiently than low-molecular-weight heparin combined with insulin therapy, but it was not superior in terms of clinical outcomes and cost [38]. Therefore, the determination of relative efficacy, meticulous risk stratification, and mortality benefits of currently used techniques, that is, insulin therapy and plasmapheresis warrant further research. Randomized clinical trials in the future may help untangle this uncertainty about the best technique in regards to the emergency management of HTGP.

## Conclusions

Patients with HTGP require urgent management as the disease presentation is particularly severe and it may result in grave complications. The use of insulin therapy with close monitoring of blood glucose levels can be an appropriate therapeutic approach, especially in cases with no availability of apheresis. This paper not only highlights the utility of insulin therapy for HTGP but also sensitizes concerned physicians to evaluate this treatment approach in larger, multicenter studies. Long-term management using pharmacological and non-pharmacological therapies, directed at maintaining the serum triglycerides within normal limits, is required to prevent recurrent attacks of HTGP.
